# Vaccine hunters and jostlers may have hurt the COVID-19 vaccination effort

**DOI:** 10.1038/s41598-022-10348-z

**Published:** 2022-04-16

**Authors:** Johanna Mollerstrom, Linda Thunström

**Affiliations:** 1 Department of Economics and ICES, George Mason University, 3434 Washington Blvd, Arlington, VA 22201 USA; 2 Research Institute of Industrial Economics, Grevgatan 34, 11215 Stockholm, Sweden; 3 Department of Economics, University of Wyoming, 1000 E. University Avenue, Laramie, WY 82071 USA

**Keywords:** Psychology, Human behaviour

## Abstract

We examine how salience of extreme actions to gain access to vaccines affect general vaccine preferences using a survey experiment conducted shortly after a limited supply of COVID-19 vaccines were made available to prioritized groups. We document that learning about people who jump the line (jostlers) or people who go through great lengths to secure left-over vaccine doses (hunters) is off-putting, and has a meaningful, negative effect on people’s vaccine preferences. Most people, however, predict the opposite—that news about extreme behavior would help the vaccination effort. If policy makers or public health authorities share these incorrect beliefs, they run the risk of implementing information policies that backfire in their effort to signal desirability of the vaccine.

## Introduction

The rapid development of COVID-19 vaccines is a phenomenal scientific achievement. The existence and availability of COVID-19 vaccines are crucial; nevertheless, the extent to which the vaccines will protect public health depends on their uptake in the population. While many people are enthusiastic about getting a COVID-19 vaccine, others remain hesitant^1–8^. A highly active research agenda therefore explores factors and policies that may encourage vaccinations^9–14^.

Several recent studies document that learning about other people’s behavior and attitudes can affect vaccine uptake. Vaccine uptake increases as a result of celebrity endorsement^15^, endorsement from one’s own party’s political elite^16^, and social norms^17^. Nevertheless, people do not only face information about the behavior of peers or authorities. While saliency of the behavior of authorities is generally covered by media, behavior of individuals among the general public typically needs to be more sensational to become salient.

As one of the most significant global health crises in our life-time, the COVID-19 pandemic offers a setting conducive to extreme behavior, in pursuit of protecting one’s own health. The perceived health threat of COVID-19 caused many people considerable anxiety, worry and stress^18–20^. The heightened health anxiety from COVID-19 could lead some people to engage in extreme, and sometimes disruptive behavior, which is harmful both to themselves and their communities^21^. At the same time, COVID-19 vaccines—widely regarded as the most effective means to control and protect from COVID-19—were initially in short supply. In this environment (in early 2021), media stories circulated about people taking extreme actions to secure a COVID-19 vaccine. In particular, the media reported stories about vaccine hunters (people not yet eligible to sign up for vaccination trying to secure left over doses^22–24^) and of rich and/or famous jostlers who used their wealth and/or influence to get vaccinated early^25–27^.

While the absolute number of hunters and jostlers is low in relation to the population, their actions got ample media attention. We are interested in whether vaccine hunters and jostlers may have impacted others’ preferences for, and enthusiasm about, the COVID-19 vaccines. On the one hand, the urgency with which hunters and jostlers wanted to get vaccinated indicated scarcity and that the vaccine was (at least to some) a highly valued good. These two factors may help increase general demand. On the other hand, the mere existence of hunters and jostlers could cause people to believe that it is so hard to get vaccinated that there is no use in even trying, thus decreasing demand. The vaccine hunters and jostlers may also be perceived as morally condemnable, and hence as people to denounce rather than copy.

## Methods

To examine the effect hunters and jostlers may have on vaccinations, we conducted two pre-registered and monetary incentivized survey experiments—a “main study” and a “follow-up study”.

### Main study

Data for the main study were collected on the Prolific platform at a time (March 22–24 of 2021) when COVID-19 vaccines were still unavailable to large groups of the adult U.S. population. We recruited a sample of Americans, representative of the population on age, gender and race/ethnicity. The survey experiment was pre-registered in the AEA registry for RCTs as AEARCTR-0007285. Informed consent was obtained from all participants, the experiment was conducted in accordance with relevant guidelines and regulations, and the experimental protocol was approved by the George Mason IRB (#1724890).

Participants were paid $1 for completion, plus any incentives earned as part of the survey. While a total of 1,503 participants answered the survey, we here focus on those (N = 1,117) who were randomized into the following treatments: i) a control treatment (participants received information about the existence of COVID-19 vaccines); ii) a “hunters” treatment (participants were additionally provided brief information that described how vaccine hunters went through great lengths to secure left-over vaccine doses); and iii) a “jostlers” treatment (providing corresponding information about how some privileged people jumped the vaccine line). The remaining 386 of participants of the total sample (N = 1,503) were randomized into a “safe” treatment. The safe treatment emphasized the safety of COVID-19 vaccines and was included to provide a benchmark for the size of any treatment effects in the hunters and the jostlers treatment. The safe treatment did, however, not impact the enthusiasm about the vaccines (most likely because our sample already have very high trust in the safety of the vaccines—more than 85% of participants believed vaccines were safe). Details on the safe treatment, its (null-)results, as well as on our participants’ trust in the vaccines, are provided in the Supplementary Information.

Specifically, participants in the control group were shown information about the existence of COVID-19 vaccines, among other things saying that “[t]he COVID-19 vaccines will decrease your risk of getting COVID-19 and of becoming seriously ill or dying […]. As the COVID-19 vaccines prevent the coronavirus from spreading and replicating, they will also help in preventing additional mutations of the virus”.

In addition to the information given in the control group, participants in the hunters and jostlers treatments saw information that described the respective phenomenon. We took care to ensure that the information contained language similar to that used in news media reporting, e.g., how vaccine supply shortages in the early spring of 2021 fueled the behavior of hunters and jostlers. Participants randomized into the hunters treatment read: “Even though the vaccines have been approved, the supply is still too low to meet the demand. This has led to the global rise of so called ‘vaccine hunters’ […]. The vaccine hunters wait for entire days outside for example grocery store pharmacies in hopes of securing left over vaccine doses (that would otherwise be discarded) at the end of the day.” Those randomized into the jostlers treatment read: “Even though the vaccines have been approved, the supply is still too low to meet the demand. This has led to a situation where, globally, the wealthy are trying to jump the line to get a COVID-19 vaccine […]. One example of this is the Canadian billionaire Rod Baker who, together with his wife, chartered a private plane and traveled to a remote region in Yukon to pose as a motel worker in order to feign being eligible for the vaccine.” Immediately after the treatment information, participants answered a short question about the main message of the paragraph. This was done to identify participants who might not be paying adequate attention, or misunderstanding the text. Such limited attention/misunderstandings were, however, uncommon: only 2.95% of participants provided an incorrect answer and our results are robust to excluding these.

Four outcome measures were assessed immediately after the treatment. First, we asked participants to state their (1) willingness to get vaccinated immediately [VAXTODAY], and (2) in two months [VAX2MONTHS] on a 1–10 scale (from definitely not being willing to definitely being willing). If participants had already received at least their first vaccine dose, these measures assessed their willingness to recommend vaccination to friends and family, using the same scale. Thereafter participants were asked whether they (3) wanted (yes/no) to get a link to general vaccine eligibility and sign-up information (for the use to self, or to friends and family) in the post survey confirmation email [VAXINFO].

The last outcome variable, which was only asked of participants who had not yet received at least a first COVID-19 vaccine dose, measured their (4) monetary valuation of a vaccine sign-up service that facilitated access to a COVID-19 vaccine. Specifically, the service provided individualized help with identifying, and signing up for, a COVID-19 vaccine appointment in the participant’s geographical vicinity once the participant became eligible (at the point of data collection, in March 2021, most adults in the U.S. were still not eligible, and many people were eager to get their vaccines as soon as they became eligible). Additional information about the vaccine sign-up service, and how it was made available to participants, is available in the Supplementary Information.

Willingness to pay for the vaccine sign-up service was elicited using a multiple-price-list, MPL^28^—participants were presented with a list of eight choice pairs. In each choice pair, participants had to decide between either getting access to the vaccine sign-up service, or instead receiving a monetary prize ($2, $5, $10, $25, $50, $75, $100 and $200 in the eight choice pairs, respectively). Participants were informed that 15 survey participants would be randomly selected and that their preferred alternative in a randomly chosen choice pair out of the eight would be implemented (i.e. they would either receive the monetary prize, or access to the vaccine sign-up service, depending on their chosen alternative in a randomly selected choice pair). Choice pairs were ordered from lowest to highest monetary amount, such that we can use the number of times a participant selected the vaccine sign-up service before switching to the monetary prize as a measure of the willingness to pay for the service [VAXHELP]. The last part of the survey assessed participants’ demographic information.

Of the 15 people randomly selected to receive their preferred alternative in the MPL, 3 participants preferred the vaccine sign-up service in the randomly selected choice pair, and 12 participants were paid a monetary price (which averaged $62).

While the sample is, by design, quota representative of the U.S. population on gender, age and race/ethnicity, it is not necessarily representative in other respects. Notably, close to 60 percent (59.4 percent, SE = 1.3) of our sample have completed at least a four year college degree, which is a higher proportion than in the general U.S. population. As education correlates positively with beliefs about vaccines in general being safe^1–9^, this might (at least partly) explain the large share of our participants believing that COVID-19 vaccines are safe.

The willingness to get vaccinated is generally high in our sample. In the control treatment, 82.6, SE = 1.9, (84.6, SE = 1.8) percent were more willing than unwilling to receive/recommend the vaccine immediately (in two months). This is at least weakly higher than corresponding shares observed in most studies who estimate willingness to get vaccinated against COVID-19 in the U.S.^1–3^. Of participants in the control treatment, 42 percent (SE = 2.49) stated that they wanted to receive information about eligibility and sign-up for COVID-19 vaccines. The willingness to pay for the vaccine sign-up service is low, however: the average participant in the control group only choose the vaccine service over the monetary prize in 0.85 (SE = 0.11) of the 8 questions, which indicates a mean WTP of less than $2.

**Figure 1:**
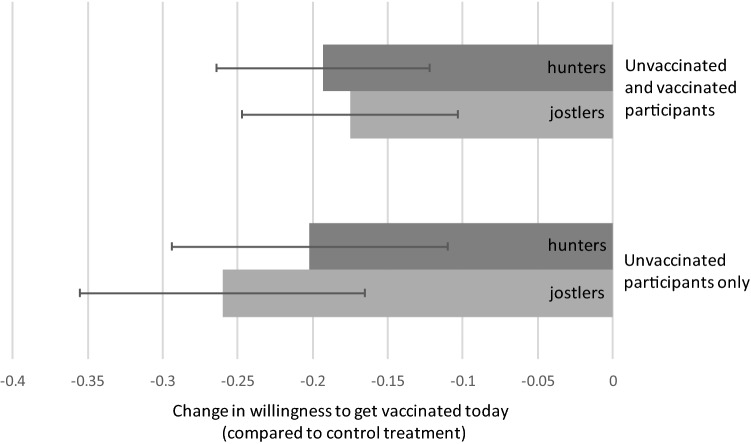
Effect of the hunters and jostlers treatments on the willingness to get vaccinated as soon as the vaccine becomes available [VAXTODAY]. The upper two bars of the hunters and jostlers treatments on all participants (Column (1) in Table 1, panel A) the lower two bars show the effect of the treatments on unvaccinated participants only (Column (1) in Table 1, panel B). Error bars denote robust standard errors.

We examined the balance of demographic and attitude variables across treatment groups by conducting 36 pairwise t-tests (two-sided) of equality of means. One test was statistically significant (*p* < 0.05): we find that the share of female participants is higher in the hunters than in the control treatment group. While this is not surprising with 36 pairwise tests, we therefore include the variable ‘female’ as a control variable in our main regression specifications reported in Fig. 1 and Table 1 (excluding this control variable does, however, not impact the results reported, or conclusions drawn).

**Table 1:** Effect of treatments on all outcome variables.

	(1)	(2)	(3)	(4)	
	vaxtoday	vax2months	vaxinfo	index	
**Main A All**					
HUNTERS	− 0.193***	− 0.169**	− 0.050	− 0.137***	
	(0.071)	(0.071)	(0.036)	(0.051)	
JOSTLERS	− 0.175**	− 0.147**	− 0.004	− 0.108**	
	(0.072)	(0.071)	(0.036)	(0.052)	
Constant	0.143***	0.135**	0.444***	0.241***	
	(0.053)	(0.053)	(0.028)	(0.039)	
Controls	Female	Female	Female	Female	
N	1117	1117	1117	1117	

	(1)	(2)	(3)	(4)	(5)
	vaxtoday	vax2months	vaxinfo	vaxhelp	index
**Main B All unvaccinated**					
HUNTERS	− 0.202**	− 0.169*	0.003	− 0.123	− 0.123*
	(0.092)	(0.092)	(0.042)	(0.150)	(0.069)
JOSTLERS	− 0.260***	− 0.214**	− 0.006	− 0.243*	− 0.181***
	(0.095)	(0.095)	(0.043)	(0.146)	(0.070)
Constant	− 0.027	− 0.018	0.411***	0.889***	0.314***
	(0.069)	(0.070)	(0.033)	(0.121)	(0.053)
Controls	Female	Female	Female	Female	Female
N	790	790	790	790	790

OLS regression results. Robust standard errors in parentheses. Significance level ***p < 0.01, **p < 0.05, *p < 0.1. Dependent variable as stated at top of columns. HUNTERS is a dummy variable equal to 1 if the participant was randomized into the HUNTERS treatment. JOSTLERS is a dummy variable equal to 1 if the participant was randomized into the JOSTLERS treatment. Panel A includes data from all participants in the CONTROL, HUNTERS and JOSTLERS treatments, regardless of vaccination status by the time of survey completion. Panel B include unvaccinated participants only.

### Follow-up study

To better understand the effect on willingness to vaccinate from the hunters and jostlers treatments that we observed in the main study, we next conducted a “follow-up study.” Data collection took place on May 19, 2021, on Prolific, and participants were 800 Americans, distinct from those who responded to the first survey. They were paid $1 for completion, plus any incentives earned as part of the study. The experimental survey used in the follow-up study elicited participants’ emotional response to the control and treatment information in the “main study,” as well as their incentivized predictions about the treatment information’s effect on the willingness to get vaccinated. This study was pre-registered in the AEA registry for RCTS as AEARCTR-0007656. Informed consent was obtained from all participants, the experiment was conducted in accordance with relevant guidelines and regulations, and the experimental protocol was approved by the George Mason IRB (#1756922-1).

## Results

We investigate the impact of information about hunters and jostlers on a total of five outcome variables. Before analyzing the effect of the treatments on our outcome variables, we standardized the outcome variables to have a mean of zero and a standard deviation of one. The first variable, VAXTODAY, measured participants’ willingness to get vaccinated as soon as a vaccine becomes available to them (or, for those who had already received at least one dose of the vaccine, the willingness to recommend friends and family to be vaccinated as soon as possible).

Our second outcome variable, VAX2MONTHS, measures the willingness to get vaccinated two months after a vaccine becomes available. Our third outcome variable, VAXINFO, assesses preferences for getting information about general vaccine eligibility and how to proceed to get a vaccine. (If participants had already received at least their first vaccine dose, we instead assessed their willingness to recommend vaccination to friends and family, as well as preferences for eligibility and sign-up information on behalf of friends and family). Finally, for our forth outcome variable, VAXHELP, participants who had not yet been vaccinated answered an incentivized question that elicited their monetary valuation of a vaccine sign-up service that would facilitate their access to a COVID-19 vaccine.

Our last outcome variable is an equally weighted index (INDEX) consisting of all applicable outcome variables. The index includes VAXTODAY, VAX2MONTHS and VAXINFO for all participants, as well as VAXHELP for the approximately 70 percent of participants who had not yet received at least the first dose of the vaccine at the time of the survey experiment. A higher value of the index indicates a higher vaccine enthusiasm for unvaccinated participants and a higher enthusiasm for others to get the vaccine for participants who already had at least a first dose of a vaccine.

Figure 1 visually depicts the effect of both the hunters and the jostlers treatment (as compared to the control), on one of our outcome variables—VAXTODAY. Table 1 shows the effect of the hunters and jostlers treatments (as compared to the control) on all five outcome variables. Panel A show the results for all participants, while panel B is restricted to participants who had not yet received at least one shot of the vaccine at the time of the survey. The results depicted in Fig. 1 and Columns (1) in panel A and B of Table 1 show that the treatments have consistently negative effects on VAXTODAY. Table 1 further shows that both treatments also have consistently negative effects on VAX2MONTHS and INDEX, while the effect on VAXINFO is close to zero and statistically insignificant. The effect on VAXHELP is also negative, and similar to the two first outcome variables in magnitude, but with less consistent statistical significance.

The results from our main study therefore imply that salience of the extreme behavior of hunters and jostlers to gain early access to a COVID-19 vaccine reduced participants’ willingness to get vaccinated and their overall enthusiasm about the vaccine. We also see indications of a similar effect on the value they ascribe to getting individualized help with securing a vaccine.

To examine whether the treatment effects are meaningful in magnitude we use the well-documented positive association between higher education and vaccine intentions (1–8) as a basis for comparison. We estimate the effect on vaccine intentions from education for our participants by running the models with the INDEX as outcome variable (model (4) in Table 1, panel A, and model (5) in Table 1, panel B), but replacing the two treatment variables with the variable “college” that takes the value 1 if the participant has completed at least four years of college, and 0 otherwise. When we base the model on the sample in panel A, the estimated coefficient for “college” is 0.343 (SE = 0.057, *p* < 0.01), and when we base it on the sample in Panel B, it is 0.328 (SE = 0.045, *p* < 0.01). Comparing the size of the estimated college coefficient to the estimated coefficients for the hunters and jostlers treatments in Table 1, we conclude that the negative effect on willingness to vaccinate by the hunters and jostlers treatments range from 31–55% of the size of the relation between higher education and the willingness to get vaccinated.

In our follow-up study, respondents first stated their experienced intensity of a given emotion (out of nine positive and negative emotions) from reading the control treatment information, and thereafter from reading either the hunter treatment or the jostler treatment information. Their emotional intensity was measured on a 1–7 Likert scale, ranging from “not at all” to “very much.” The largest changes in emotions from both the hunters and the jostlers treatment information were a decrease in happiness ( − 3.18, SE = 0.096 in the hunters, and − 3.67, SE = 0.101 in the jostlers treatment), an increase in anger (1.58, SE = 0.096, and 3.62, SE = 0.107, respectively), and an increase in sadness (1.70, SE = 0.097, and 2.28, SE = 0.109, respectively). In addition, the jostlers treatment triggered a prominent increase in feeling disgusted (3.84, SE = 0.109). This decrease in happiness and increase in negative emotions could potentially explain, at least in part, the drop in willingness to get vaccinated caused by the treatments.

Participants in the follow-up study were then introduced to the set-up of our main study and asked to make an incentivized prediction about the directional effect of the treatments on vaccine enthusiasm. Interestingly, the most common prediction was that the willingness to get vaccinated would increase, with 52.7% (51.0%) predicting this result for the hunters (jostlers) treatment. Only 9.5% (9.3%) predicted, accurately, that the willingness to get vaccinated would decrease from the treatments. To the extent that actual vaccine hunters and jostlers share in our participants’ predictions, our results indicate that the negative spillover effect from their extreme vaccine efforts might have been unexpected to them. Indeed, hustlers and jostlers may have actually believed that their actions could help increase the general willingness to get vaccinated (perhaps by signaling vaccine desirability), while failing to account for a counteracting, negative effect on vaccine intentions.

## Discussion

While previous studies show that behavior and attitudes of celebrities, endorsed elites and peers may affect public health efforts in a major health crisis^15–17^, our results show that behavior by single individuals amongst the general public may similarly matter, when their efforts to self-protect are rather extreme and thus become salient to others, e.g., gain attention from media. We find that individual extreme efforts to self-protect can evoke immediate negative emotions in others and therefore may deter others from engaging in the same type of self-protection. More precisely, we find that salience of extreme actions of some individuals, i.e., hunters and jostlers, to secure a COVID-19 vaccine might have negatively affected other people’s enthusiasm for getting vaccinated and/or willingness to recommend others to get a COVID-19 vaccine. We encourage future research to examine the robustness of our results across samples, contexts and types of public health efforts.

## Supplementary Information

Supplementary Information.

## Data Availability

All data, code and experimental survey instruments developed for this study are publicly available at openICPSR under Number 150081.
