# Supplementary material for: Vaccine hunters and jostlers may have hurt the COVID-19 vaccination effort
Source: Sci Rep. 2022 Apr 16;12:6382. doi: 10.1038/s41598-022-10348-z (PMC9013183; doi:10.1038/s41598-022-10348-z)
Supplement: Supplementary file 1 — Supplementary Information. [file 41598_2022_10348_MOESM1_ESM.pdf]

Supplementary information

Vaccine hunters and jostlers may have hurt the COVID-19 vaccination effort

Johanna Mollerstrom[1], Linda Thunström[2]

[1] Corresponding author. Department of Economics and ICES, George Mason University, 3434 Washington Blvd, Arlington 22201 Virginia, USA. Research Institute of Industrial Economics, Grevgatan 34, 11215 Stockholm, Sweden. Email: jmollers@gmu.edu.

[2] Department of Economics, University of Wyoming, 1000 E. University Avenue, Laramie 82071, Wyoming, USA.

Table S1: Descriptive Statistics of participants in Main study

	CONTROL	HUNTERS	JOSTLERS
Female	0.455 (0.025)	0.527 (0.027)	0.497 (0.026)
Age	45.6 (0.807)	45.7 (0.857)	46.0 (0.831)
Black	0.119 (0.016)	0.130 (0.018)	0.136 (0.018)
Asian	0.088 (0.014)	0.057 (0.012)	0.060 (0.012)
Completed 4 year college or more	0.571 (0.025)	0.601 (0.026)	0.601 (0.026)
Had covid	0.061 (0.012)	0.065 (0.013)	0.063 (0.013)
Know someone who had covid	0.793 (0.020)	0.759 (0.023)	0.788 (0.021)
Knew someone who died of covid	0.308 (0.023)	0.292 (0.024)	0.318 (0.024)
Worry about getting COVID	0.692 (0.023)	0.748 (0.023)	0.693 (0.024)
Believe COVID-19 vaccines are safe	0.891 (0.016)	0.858 (0.019)	0.864 (0.018)
Believe masks are important to combat pandemic	0.876 (0.017)	0.884 (0.017)	0.886 (0.017)
Received at least first vaccine dose	0.290 (0.023)	0.266 (0.024)	0.321 (0.024)
N	396	353	368

Means with SE in parentheses. Age in years, all other variables measured as share of sample. Worry about getting covid are those who say that they worry slightly, moderately or strongly about being infected with COVID-19. Believe masks are important are those who say that they believe masks are slightly, moderately or strongly important to combat the COVID-19 pandemic. A person is categorized as believing that the vaccines are safe is they answer that they think that the COVID vaccines are fairly or very safe (as opposed to fairly or very unsafe).

Vaccine signup service – additional implementation details

Per the user agreement of Prolific, researchers cannot ask for a survey respondent's name, or other identifying information. The vaccine appointment sign-up service hence had to be implemented under this constraint. All contact between the team and the three respondents who were 1) randomly selected to receive a prize, and 2) had chosen the vaccine signup service in the randomly chosen choice pair item, therefore took place using the respondents' anonymous prolific email address.

Each of the three respondents were first sent general questions that helped the researchers identify their eligibility status, and their geographical location in enough detail to be able to help identify an appointment, but not so detailed as to be identifying. Thereafter email conversations between the individual respondents and the team took place, in which eligibility was determined and conveyed, and help was provided to make sure they got a vaccine appointment as soon as possible in their geographical vicinity.

In one case this involved simply verifying that the respondent who worked as an elementary school tutor was in the same eligibility group as elementary school teachers in the relevant state, and then direct them to their state's central appointment making webpage where they could immediately make an appointment. In the other two cases the service consistent of identifying when the respondents became eligible for COVID-19 vaccination, alert them to pharmacies with vaccine in stock close to the their homes in their respective state, and sending the links to sign up. In all three cases the respondents reportedly were successful in securing appointments after having received this assistance.

The “safe” treatment

A fourth treatment, not discussed in detail in the main text, showed participants a paragraph which highlights the fact that all vaccines move through an approval process ensuring that they are safe to use. The information shown to the N=368 respondents randomized into the “safe” treatment was:

“All vaccines in the U.S. must go through pre-clinical and clinical development stages before they are approved by the U.S. Food and Drug Administration (FDA). The COVID-19 vaccine process has been expedited by the FDA to make a vaccine available to the public to combat the pandemic. The FDA is streamlining certain steps of the process and allowing some early phases of clinical development to be done simultaneously, but all traditional safety and effectiveness standards are being maintained.”

This treatment was included in order to establish the hypothesized significantly positive difference in willingness to get vaccinated between the safe and the control treatment as a baseline, against which the magnitudes of other treatment effects could be compared. The safe treatment did, however, not impact the willingness to get vaccinated: If we add the 386 observations from the safe treatment to the data, and rerun regression specification (1)-(4) from Table 1A also including a dummy variable for the safe treatment, the coefficient on safe is -0.075 (SE=0.050), -0.119 (0.069), -0.119 (0.069) and 0.014 (0.035), respectively (remaining results presented in the manuscript are unaffected). None of the coefficients on safe are statistically significant.

The fact that the respondents in our sample did not react significantly to the safe treatment could potentially be at least partly explained by the very high trust in the safety of the vaccine before treatment (see Table S1). Instead of using a non-significant treatment effect as a comparison, we instead use the well-studied positive relation between amount of education and willingness to get vaccinated, as outlined in the main text.

Survey Materials for Main Study

Start of Block: Prolific ID

Thank you for participating in our study. We estimate that this study will take about 5 minutes to complete. After you have finished, you will receive a completion code. Please return to Prolific and enter the completion code in the space provided.

To receive your bonus payment, you must enter your Prolific ID into the box below and then click to continue.

Enter your ID here:

____

End of Block: Prolific ID

Start of Block: Consent

You will receive **$1 for completing the survey**. In addition to that, you can earn **an additional bonus**. The rules for the additional bonus are described later. We will now go through the instructions. Please read them carefully. You are only eligible for payment if you adhere to the instructions. As established researchers on Prolific, we promise that the information in this survey is truthful and accurate and we will send you the money you earn in the survey. If you have any questions about this research, please feel free to email us at prolific.survey.research@gmail.com. Please press the NEXT button to proceed.

Page Break

CONSENT FORM

RESEARCH PROCEDURES This research is being conducted to study individual opinions and decision making. If you agree to participate in this study, you will make decisions using your computer or other device. At the end of the study, you will complete a questionnaire. The study will take around 5 minutes.

RISKS
There is always a slight chance that someone might feel upset after completing the survey, however it is important to know that there are no expected risks or negative effects associated with your involvement. Please note that if you do feel upset and would like to speak with someone, you can contact the National Alliance on Mental Illness (NAMI) at 1-800-950-NAMI (6264) or info@nami.org.

BENEFITS
There are no benefits to you as a participant other than to further research in economic decision making.

CONFIDENTIALITY
The data in this research will be confidential. Names and other personal information will not be placed on surveys or other research data. You will make all of your decisions anonymously. Your Prolific identification is only collected for purposes of compensation and reimbursement. Such identification will be kept separate from any collected data. While it is understood that no computer transmission can be perfectly secure, reasonable efforts will be made to protect the confidentiality of your transmission. The de-identified data could be used for future research without additional consent from participants. The Institutional Review Board (IRB) committee that monitors research on human subjects may inspect study records during internal auditing procedures and are required to keep all information confidential.

PARTICIPATION
You must be 18 years or older, reside in the US and have an active Prolific account to take part in the study. Your participation is voluntary, and you may withdraw from the study at any time and for any reason. If you decide not to participate or if you withdraw from the study, there is no penalty or loss of benefits to which you are otherwise entitled. There are no costs to you or any other party.

You will be eligible to receive the $1 reward plus potential bonus earnings from this study only if you adhere to the instructions. In particular, you will need to copy the completion code at the end of the survey into the appropriate box on the Prolific webpage. Under the U.S. federal tax law you may have individual responsibilities for disclosing the dollar value of the incentive received on this study.

CONTACT
This research is being conducted by researchers at George Mason University and the University of Wyoming. The researchers may be reached at prolific.survey.research@gmail.com and at (757)-514-1303 for questions or to report a research related problem. You may contact the George Mason University Institutional Review Board (IRB) Office at 703-993-4121, or at irb@gmu.edu if you have questions or comments regarding your rights as a participant in the research (IRBNet number for this study is 1724890-1]).

CONSENT
I confirm that I have read this form, all of my questions have been answered, and I agree to participate in this study.

○ Yes, I agree to participate in this study

○ No, I do not want to participate in this study

End of Block: Consent

Start of Block: COVID info

On March 11, 2020 the World Health Organization (WHO) declared COVID-19 a global pandemic. The novel coronavirus (SARS-CoV-2), is highly infectious and causes the COVID-19 disease.

People who contract the novel coronavirus (SARS-CoV-2), may or may not experience symptoms of COVID-19, i.e., they may be symptomatic or non-symptomatic. Most infected people will develop mild to moderate illness. Symptoms vary from mild (e.g., fever, cough) to severe (e.g., difficulty breathing, chest pain), or even death (see https://www.who.int/health-topics/coronavirus).
COVID-19 poses a substantial health risk particularly to elderly and those with preexisting medical conditions.

As of March 12, the United States has had 29,052,862 total cases and 527,726 deaths as a result of COVID-19 (https://covid.cdc.gov/covid-data-tracker/#cases_totalcases). It is still unclear to what extent a person who has been infected is protected from contracting the virus again.

○ I confirm that I read the above text

○ I did not read the above text

End of Block: COVID info

Start of Block: COVID experiences

Have you tested positive for COVID-19 in the past?

○ Yes, I have

○ No, I have not

Has anyone you know tested positive for COVID-19 in the past?

○ Yes, a family member has tested positive.

○ Yes, someone in my social network has tested positive.

○ No, I do not know anyone who has tested positive.

	Yes	No
I know someone who died from COVID-19.	○	○
I know someone who was very sick from COVID-19.	○	○

Please state the extent to which you agree or disagree with the below statements about the impact COVID-19 has had on you.

	Strongly agree	Agree	Somewhat agree	Neither agree nor disagree	Somewhat disagree	Disagree	Strongly disagree
My finances have been negatively impacted by COVID-19.	○	○	○	○	○	○	○
I have lost my job due to COVID-19.	○	○	○	○	○	○	○
My social life has been negatively impacted by COVID-19.	○	○	○	○	○	○	○
My mental health has been negatively impacted by COVID-19.	○	○	○	○	○	○	○
My physical health has been negatively impact by COVID-19.	○	○	○	○	○	○	○

End of Block: COVID experiences

Start of Block: COVID perceptions

At this point, how serious do you think the health consequences to you would be from getting COVID-19?

- Very serious
- Serious
- Somewhat serious
- Not serious

At this point, how serious do you think the financial consequences to you would be from getting COVID-19?

- Very serious
- Serious
- Somewhat serious
- Not serious

Do you think that the approved COVID-19 vaccines are safe?

- Yes, very safe
- Yes, fairly safe
- No, fairly unsafe
- No, very unsafe

Please state the extent to which you agree or disagree with the below statements.

	Strongly disagree	Moderately disagree	Slightly disagree	Neutral	Slightly agree	Moderately agree	Strongly agree
I worry about getting infected by COVID-19.	○	○	○	○	○	○	○
I worry about my mental health during the pandemic.	○	○	○	○	○	○	○
I worry about the healthcare system becoming overwhelmed.	○	○	○	○	○	○	○
I worry about my financial security.	○	○	○	○	○	○	○
I worry about my local economy.	○	○	○	○	○	○	○
I worry about the United States economy.	○	○	○	○	○	○	○
Social distancing is important to stop the spread of COVID-19.	○	○	○	○	○	○	○
Wearing a face mask in public is important to stop the spread of COVID-19.	○	○	○	○	○	○	○

End of Block: COVID perceptions

Start of Block: Treatment 1: Vaccine billionaires

There are now several COVID-19 vaccines available. The COVID-19 vaccines will decrease your risk of getting COVID-19 and of becoming seriously ill or dying from COVID-19. They might also prevent you from spreading the coronavirus to others, and contribute to herd immunity. As the COVID-19 vaccines prevent the coronavirus from spreading and replicating, they will also help in preventing additional mutations of the virus.

Even though vaccines have been approved, the supply is still too low to meet the demand. This has led to a situation where, globally, the wealthy are trying to jump the line to get a COVID-19 vaccine amid chaotic rollouts and supply shortages. One of many examples of this is the Canadian billionaire Rod Baker who, together with his wife, chartered a private plan and traveled to a remote region in Yukon to pose as a motel worker in order to feign being eligible for the vaccine.

Which of the following is a main point of the text above?

- ○ That some people use various strategies to get the vaccine before technically being eligible.
- ○ The economic consequences for society of the COVID-19 pandemic.
- ○ None of the above

End of Block: Treatment 1: Vaccine billionaires

Start of Block: Treatment 2: Vaccine hunters

There are now several COVID-19 vaccines available. The COVID-19 vaccines will decrease your risk of getting COVID-19 and of becoming seriously ill or dying from COVID-19. They might also prevent you from spreading the coronavirus to others, and contribute to herd immunity. As the COVID-19 vaccines prevent the coronavirus from spreading and replicating, they will also help in preventing additional mutations of the virus.

Even though vaccines have been approved, the supply is still too low to meet the demand. This has led to the global rise of so called "vaccine hunters," ordinary people who are not yet eligible for the vaccine but are nevertheless determined to get the vaccine amid chaotic rollouts and supply shortages. The vaccine hunters wait for entire days outside for example grocery store pharmacies in hopes of securing left over vaccine doses (that would otherwise be discarded) at the end of the day.

Which of the following is a main point of the text above?

- That some people use various strategies to get the vaccine before technically being eligible.
- The economic consequences for society of the COVID-19 pandemic.
- None of the above

End of Block: Treatment 2: Vaccine hunters

Start of Block: Treatment 3: Vaccine scientific

There are now several COVID-19 vaccines available. The COVID-19 vaccines will decrease your risk of getting COVID-19 and of becoming seriously ill or dying from COVID-19. They might also prevent you from spreading the coronavirus to others, and contribute to herd immunity. As the COVID-19 vaccines prevent the coronavirus from spreading and replicating, they will also help in preventing additional mutations of the virus..

All vaccines in the U.S. must go through pre-clinical and clinical development stages before they are approved by the U.S. Food and Drug Administration (FDA). The COVID-19 vaccine process has been expedited by the FDA to ensure a vaccine is made available to the public in a timely manner to combat the pandemic. The FDA is streamlining certain steps of the process and allowing some early phases of clinical development to be done simultaneously, but all traditional safety and effectiveness standards are being maintained.

Which of the following is a main point of the text above?

- COVID-19 vaccines are tested for safety before they become available to the public.
- The economic consequences for society of the COVID-19 pandemic.
- None of the above

End of Block: Treatment 3: Vaccine scientific

Start of Block: Treatment 4: Control

There are now several COVID-19 vaccines available. The COVID-19 vaccines will decrease your risk of getting COVID-19 and of becoming seriously ill or dying from COVID-19. They might also prevent you from spreading the coronavirus to others, and contribute to herd immunity. As the COVID-19 vaccines prevent the coronavirus from spreading and replicating, they will also help in preventing additional mutations of the virus.

Which of the following is a main point of the text above?

- ○ There now exist vaccines for COVID-19.
- ○ The economic consequence for society of the COVID-19 pandemic.
- ○ None of the above

End of Block: Treatment 4: Control

Start of Block: Vaccinated?

Have you gotten at least the first dose of a COVID-19 vaccine?

- ○ Yes
- ○ No

End of Block: Vaccinated?

Start of Block: Outcome variables - vaccinated

If an approved COVID-19 vaccine would be available to a close family member or friend **today**, would you recommend them to receive it?

- ○ 1 (Definitely no)
- ○ 2
- ○ 3
- ○ 4
- ○ 5
- ○ 6
- ○ 7
- ○ 8
- ○ 9
- ○ 10 (Definitely yes)

If they had not previously gotten a vaccine, and an approved COVID-19 vaccine becomes available to a close family member or friend **in two months**, would you recommend them to receive it?

- ○ 1 (Definitely no)
- ○ 2
- ○ 3
- ○ 4
- ○ 5
- ○ 6
- ○ 7
- ○ 8
- ○ 9
- ○ 10 (Definitely yes)

Page Break

When you have finished the survey, we will send you a confirmation message on Prolific. Would you like us to include a link to a website with more information about when people will be eligible to receive the vaccine in different states in the United States, and information about how to schedule an appointment? You could then forward this link to family and friends who have not yet gotten the vaccine.

- Yes
- No

Page Break

To what extent do you trust in the ability of local and federal decision makers in the U.S. to handle the COVID-19 pandemic?

- 1 (No trust at all)
- 2
- 3
- 4
- 5
- 6
- 7
- 8
- 9
- 10 (Extremely high trust)

End of Block: Outcome variables - vaccinated

Start of Block: Outcome variables - not vaccinated

If an approved COVID-19 vaccine would be available to you **today**, would you like to receive it?

- ○ 1 (Definitely no)
- ○ 2
- ○ 3
- ○ 4
- ○ 5
- ○ 6
- ○ 7
- ○ 8
- ○ 9
- ○ 10 (Definitely yes)

If you had not previously gotten a vaccine, and an approved COVID-19 vaccine becomes available to you **in two months**, would you like to receive it?

- 1 (Definitely no)
- 2
- 3
- 4
- 5
- 6
- 7
- 8
- 9
- 10 (Definitely yes)

Page Break

When you have finished the survey, we will send you a confirmation message on Prolific. Would you like us to include a link to a website where you can get more information about when you will be eligible to receive the vaccine in different states in the United States, and information about how to schedule an appointment?

- Yes
- No

Page Break

Even if you are eligible for the vaccine, the shortage of vaccine doses unfortunately often makes it tricky and time consuming both to identify, and sign up for, an appointment to get the vaccine

(see e.g., Washington Post; Fox News).

As an additional thank you for responding to our survey, we will randomly select 15 respondents who will **get one of two prices**: 1) A monetary prize (transferred to you as a bonus on Prolific) or 2) Help from trained assistants to identify an available appointment near you and assist you with the signup process, as soon as you become eligible for vaccination in your state.

In the table below, please select in each row whether you would like the monetary prize, or access to the vaccine signup service.

If you are one of the 15 selected respondents who will receive a prize, one of the rows in the table will be randomly selected for you, and your choice in that row will determine which prize you will receive.

If you selected a monetary prize, it will be transferred to you via Prolific. If you selected access to the vaccine signup service, you will get information about how to email the assistants who will provide the service using your anonymous prolific email (the service will not ask you to disclose any identifying information).

	1	2	
Monetary prize of $2	○	○	Access to vaccine signup service
Monetary prize of $5	○	○	Access to vaccine signup service
Monetary prize of $10	○	○	Access to vaccine signup service
Monetary prize of $25	○	○	Access to vaccine signup service
Monetary prize of $50	○	○	Access to vaccine signup service
Monetary prize of $75	○	○	Access to vaccine signup service
Monetary prize of $100	○	○	Access to vaccine signup service
Monetary prize of $200	○	○	Access to vaccine signup service

Page Break

To what extent do you trust in the ability of local and federal decision makers in the U.S. to handle the COVID-19 pandemic?

- ◯ 1 (No trust at all)
- ◯ 2
- ◯ 3
- ◯ 4
- ◯ 5
- ◯ 6
- ◯ 7
- ◯ 8
- ◯ 9
- ◯ 10 (Extremely high trust)

End of Block: Outcome variables - not vaccinated

Start of Block: Demographics: personality

How do you see yourself: Are you generally a person who is fully prepared to take risks or do you try to avoid taking risks?

- ○ 1(Not at all willing to take risks)
- ○ 2
- ○ 3
- ○ 4
- ○ 5
- ○ 6
- ○ 7
- ○ 8
- ○ 9
- ○ 10 (very willing to take risks)

Do you consider yourself a competitive person? Please rate on a scale of 1 to 10

- 1(Not competitive at all)
- 2
- 3
- 4
- 5
- 6
- 7
- 8
- 9
- 10 (Extremely competitive)

End of Block: Demographics: personality

Start of Block: Demographics basic

When it comes to social issues, how liberal or conservative are you?

- 1(Very liberal)
- 2
- 3
- 4
- 5
- 6
- 7
- 8
- 9 (Very conservative)

When it comes to economic issues, how liberal or conservative are you?

- 1(Very liberal)
- 2
- 3
- 4
- 5
- 6
- 7
- 8
- 9 (Very conservative)

Do you generally think of yourself as a Democrat, Republican, Independent, or something else?

- Democrat
- Republican
- Independent
- Other

Page Break

What is your gender?

- ○ Female
- ○ Male
- ○ Other

---

*

What is your age in years?

____

What is the highest level of education you have completed?

- ○ Less than High School
- ○ High School / GED
- ○ Some college
- ○ 2 year degree
- ○ 4 year degree
- ○ Professional degree
- ○ Doctorate

Which of these best describes your race/ethnicity?

- ○ White/Caucasian
- ○ Black/ African American
- ○ Hispanic/ Latino
- ○ Asian
- ○ Native American
- ○ Pacific Islander
- ○ Other

What was your total income last year? Take into account all your sources of income, including scholarships, health benefits, fringe benefits, and others. Please note that this is your personal income, not the income of your household.

- ○ Less than $10,000
- ○ $10,000 to $20,000
- ○ $20,000 to $30,000
- ○ $30,000 to $40,000
- ○ $40,000 to $50,000
- ○ $60,000 to $70,000
- ○ $70,000 to $80,000
- ○ $90,000 to $100,000
- ○ Over $100,000

In which state do you currently reside?

▼ Alabama ... I do not reside in the United States

End of Block: Demographics basic

Survey Materials for Follow-up Study

Start of Block: Prolific ID

Thank you for participating in our survey. We estimate that this survey will take about 5 minutes to complete. After you have finished, you will receive a completion code. Please return to Prolific and enter the completion code in the space provided.

To receive your bonus payment, you must enter your Prolific ID into the box below and then click to continue.

Enter your ID here:

____

End of Block: Prolific ID

Start of Block: Consent

You will receive **$1 for completing the survey**. **You may get an additional $0.50**, depending on your choices in the survey.

We will now go through the instructions. Please read them carefully. Please note that you are not allowed to restart the survey once you have started it.

As established researchers on Prolific, we promise that the information in this survey is truthful and accurate and we will send you the money you earn in the survey. If you have any questions about this research, please feel free to email us at prolific.survey.research@gmail.com.

Please press the NEXT button to proceed.

Page Break

CONSENT FORM

RESEARCH PROCEDURES
This research aims to study individual opinions. If you agree to participate in this study, you will offer your opinions using your computer or other device. You will be asked to provide answers a number of questions, including an open ended question, for which we require that you provide an answer that is 1-3 sentences long. The study will take around 5 minutes to complete.

RISKS
There is always a slight chance that someone might feel upset after completing the survey, however it is important to know that there are no expected risks or negative effects associated

with your involvement. Please note that if you do feel upset and would like to speak with someone, you can contact the National Alliance on Mental Illness (NAMI) at 1-800-950-NAMI (6264) or info@nami.org.

BENEFITS

There are no benefits to you as a participant other than to further research in economic decision making.

CONFIDENTIALITY

The data in this research will be confidential. Names and other personal information will not be placed on surveys or other research data. You will make all of your decisions anonymously. Your Prolific identification is only collected for purposes of compensation and reimbursement. Such identification will be kept separate from any collected data. While it is understood that no computer transmission can be perfectly secure, reasonable efforts will be made to protect the confidentiality of your transmission. The de-identified data could be used for future research without additional consent from participants. The Institutional Review Board (IRB) committee that monitors research on human subjects may inspect study records during internal auditing procedures and are required to keep all information confidential.

PARTICIPATION

You must be 18 years or older, reside in the US and have an active Prolific account to take part in the study. Your participation is voluntary, and you may withdraw from the study at any time and for any reason. If you decide not to participate or if you withdraw from the study, there is no penalty or loss of benefits to which you are otherwise entitled. There are no costs to you or any other party.

You will be eligible to receive the $1 reward plus potential bonus earnings ($0.50) from this study only if you adhere to the instructions. In particular, you will need to copy the completion code at the end of the survey into the appropriate box on the Prolific webpage. Under the U.S. federal tax law you may have individual responsibilities for disclosing the dollar value of the incentive received on this study. You are only eligible to participate in the survey once.

CONTACT

This research is being conducted by researchers at George Mason University and the University of Wyoming. The researchers may be reached at prolific.survey.research@gmail.com and at (757)-514-1303 for questions or to report a research related problem. You may contact the George Mason University Institutional Review Board (IRB) Office at 703-993-4121, or at irb@gmu.edu if you have questions or comments regarding your rights as a participant in the research (IRBNet number for this study is 1756922-1).

CONSENT

I confirm that I have read this form, all of my questions have been answered, and I agree to participate in this study.

- ◯ Yes, I agree to participate in this study
- ◯ No, I do not want to participate in this study

End of Block: Consent

Start of Block: COVID info

X→

On March 11, 2020 the World Health Organization (WHO) declared COVID-19 a global pandemic. The novel coronavirus (SARS-CoV-2), is highly infectious and causes the COVID-19 disease.

People who contract the novel coronavirus (SARS-CoV-2), may or may not experience symptoms of COVID-19, i.e., they may be symptomatic or non-symptomatic. Most infected people will develop mild to moderate illness. Symptoms vary from mild (e.g., fever, cough) to severe (e.g., difficulty breathing, chest pain), or even death (see https://www.who.int/health-topics/coronavirus).
COVID-19 poses a substantial health risk particularly to elderly and those with preexisting medical conditions.

As of April 28 2021, the United States has had 32,571,814 total cases and 579,366 deaths as a result of COVID-19 (https://covid.cdc.gov/covid-data-tracker/#cases_totalcases). It is still unclear to what extent a person who has been infected is protected from contracting the virus again.

- ◯ I confirm that I read the above text
- ◯ I did not read the above text

End of Block: COVID info

Start of Block: Both T1 and T2: Control

In this survey, we will describe a previously conducted research study to you. All participants in the research study read the following <u>first piece of information</u>:

"There are now several COVID-19 vaccines available. The COVID-19 vaccines will decrease your risk of getting COVID-19 and of becoming seriously ill or dying from COVID-19. They might also prevent you from spreading the coronavirus to others, and contribute to herd immunity. As the COVID-19 vaccines prevent the coronavirus from

spreading and replicating, they will also help in preventing additional mutations of the virus."

Which of the following is a main point of this first piece of information?

- There now exist vaccines for COVID-19.
- The economic consequence for society of the COVID-19 pandemic.
- None of the above

Does the first piece of information above make you feel...

	(Not at all) 1	2	3	4	5	6	(Very much) 7
Happy	○	○	○	○	○	○	○
Sad	○	○	○	○	○	○	○
Angry	○	○	○	○	○	○	○
Anxious	○	○	○	○	○	○	○
Disgusted	○	○	○	○	○	○	○
Indignated	○	○	○	○	○	○	○
Envious	○	○	○	○	○	○	○
Confused	○	○	○	○	○	○	○
Bored	○	○	○	○	○	○	○

End of Block: Both T1 and T2: Control

Start of Block: T1: Jostling billionaires

As stated on the previous page, all participants in the research study read the following first piece of information: "There are now several COVID-19 vaccines available. The COVID-19 vaccines will decrease your risk of getting COVID-19 and of becoming seriously ill or dying from COVID-19. They might also prevent you from spreading the coronavirus to others, and contribute to herd immunity. As the COVID-19 vaccines prevent the coronavirus from spreading and replicating, they will also help in preventing additional mutations of the virus."

Some participants in the research study thereafter read a second piece of information:

"Even though vaccines have been approved, the supply is still too low to meet the demand. This has led to a situation where, globally, the wealthy are trying to jump the line to get a

COVID-19 vaccine amid chaotic rollouts and supply shortages. One of many examples of this is the Canadian billionaire Rod Baker who, together with his wife, chartered a private plan and traveled to a remote region in Yukon to pose as a motel worker in order to feign being eligible for the vaccine."

Which of the following is a main point of the second piece of information?

- ◯ That some people used various strategies to get the vaccine before technically being eligible.
- ◯ The economic consequence for society of the COVID-19 pandemic.
- ◯ None of the above

Does the second piece of information make you feel...

	(Not at all) 1	2	3	4	5	6	(Very much) 7
Happy	○	○	○	○	○	○	○
Sad	○	○	○	○	○	○	○
Angry	○	○	○	○	○	○	○
Anxious	○	○	○	○	○	○	○
Disgusted	○	○	○	○	○	○	○
Indignated	○	○	○	○	○	○	○
Envious	○	○	○	○	○	○	○
Confused	○	○	○	○	○	○	○
Bored	○	○	○	○	○	○	○

Page Break

You have now seen the following pieces of information:

First:
"There are now several COVID-19 vaccines available. The COVID-19 vaccines will decrease your risk of getting COVID-19 and of becoming seriously ill or dying from COVID-19. They might also prevent you from spreading the coronavirus to others, and contribute to herd immunity. As the COVID-19 vaccines prevent the coronavirus from spreading and replicating, they will also help in preventing additional mutations of the virus."

Second:
"Even though vaccines have been approved, the supply is still too low to meet the demand. This has led to a situation where, globally, the wealthy are trying to jump the line to get a COVID-19 vaccine amid chaotic rollouts and supply shortages. One of many examples of this is the Canadian billionaire Rod Baker who, together with his wife, chartered a private plan and traveled to a remote region in Yukon to pose as a motel worker in order to feign being eligible for the vaccine."

The research study compared the willingness to get vaccinated against Covid-19 (and to recommend friends and family to get vaccinated), for the people who ONLY read the first piece of information, to the people who ALSO read the second piece of information. 1500 U.S. adults participated in the study. The study was conducted in early spring 2021, when COVID-19 vaccines in the U.S. were in short supply, such that it was difficult to get vaccinated. Also, at that time, only prioritized groups were eligible to get a COVID-19 vaccine.

We now ask you to guess the results of the study. One of the three alternatives is the actual result, and if you guess it correctly, we will transfer you an additional $0.50.

○ I believe people in the study were MORE willing to get vaccinated after seeing both pieces of information (compared to when they saw only the first piece of information)

○ I believe people in the study were LESS willing to get vaccinated after seeing both pieces of information (compared to when they saw only the first piece of information)

○ I believe that seeing both pieces of information DID NOT CHANGE the willingness to get vaccinated of people in the study (compared to when they say only the first piece of information)

Page Break

The actual result in the study was that people who also read the second piece of information (about some rich people jumping the line to get vaccinated) were LESS willing to get vaccinated (and less willing to recommend the vaccine to others), compared to those who did not get this piece of information. (Please do not share this result with anyone who has a prolific-account, in order to preserve the integrity of this study.)

Why do you think people became LESS willing to vaccinate when they learned that some rich people were jumping the line to get the vaccine? Please answer with at least 1 and at most 3 sentences below.

____

____

____

____

____

Page Break

How **likely do you think it is that any of the below reasons explain why** people were less willing to get vaccinated against COVID-19 when they learned that some rich people jumped the line to get the vaccine?

Learning about some rich people jumping the line for vaccines makes people...

	Very unlikely	Unlikely	Neither unlikely/likely	Likely	Very likely
...think there is no use in trying to get a vaccine	○	○	○	○	○
...care less about getting vaccinated in order to protect their own health	○	○	○	○	○
...care less about getting vaccinated in order to protect other people's health	○	○	○	○	○
...care less about doing "the right thing" (in this case getting vaccinated)	○	○	○	○	○
...think vaccines are more unsafe	○	○	○	○	○
...think it is less risky to get sick from COVID-19	○	○	○	○	○

End of Block: T1: Jostling billionaires

Start of Block: T2: Vaccine hunters

As stated on the previous page, all participants in the research study read the following first

piece of information: "There are now several COVID-19 vaccines available. The COVID-19 vaccines will decrease your risk of getting COVID-19 and of becoming seriously ill or dying from COVID-19. They might also prevent you from spreading the coronavirus to others, and contribute to herd immunity. As the COVID-19 vaccines prevent the coronavirus from spreading and replicating, they will also help in preventing additional mutations of the virus."

Some participants in the research study thereafter read a second piece of information:

"Even though vaccines have been approved, the supply is still too low to meet the demand. This has led to the global rise of so called "vaccine hunters," ordinary people who are not yet eligible for the vaccine but are nevertheless determined to get the vaccine amid chaotic rollouts and supply shortages. The vaccine hunters wait for entire days outside for example grocery store pharmacies in hopes of securing left over vaccine doses (that would otherwise be discarded) at the end of the day."

Which of the following is a main point of the second piece of information?

- ○ That some people used various strategies to get the vaccine before technically being eligible.
- ○ The economic consequence for society of the COVID-19 pandemic.
- ○ None of the above

Does the second piece of information make you feel...

	(Not at all) 1	2	3	4	5	6	(Very much) 7
Happy	○	○	○	○	○	○	○
Sad	○	○	○	○	○	○	○
Angry	○	○	○	○	○	○	○
Anxious	○	○	○	○	○	○	○
Disgusted	○	○	○	○	○	○	○
Indignated	○	○	○	○	○	○	○
Envious	○	○	○	○	○	○	○
Confused	○	○	○	○	○	○	○
Bored	○	○	○	○	○	○	○

Page Break

You have now seen the following pieces of information:

First:
"There are now several COVID-19 vaccines available. The COVID-19 vaccines will decrease your risk of getting COVID-19 and of becoming seriously ill or dying from COVID-19. They might also prevent you from spreading the coronavirus to others, and contribute to herd immunity. As the COVID-19 vaccines prevent the coronavirus from spreading and replicating, they will also help in preventing additional mutations of the virus."

Second:
"Even though vaccines have been approved, the supply is still too low to meet the demand. This has led to the global rise of so called "vaccine hunters," ordinary people who are not yet eligible for the vaccine but are nevertheless determined to get the vaccine amid chaotic rollouts and supply shortages. The vaccine hunters wait for entire days outside for example grocery store pharmacies in hopes of securing left over vaccine doses (that would otherwise be discarded) at the end of the day."

The research study compared the willingness to get vaccinated against Covid-19 (and to recommend friends and family to get vaccinated), for the people who ONLY read the first piece of information, to the people who ALSO read the second piece of information. 1500 U.S. adults participated in the study. The study was conducted in early spring 2021, when COVID-19 vaccines in the U.S. were in short supply, such that it was difficult to get vaccinated. Also, at that time, only prioritized groups were eligible to get a COVID-19 vaccine.

We now ask you to guess the results of the study. One of the three alternatives is the actual result, and if you guess it correctly, we will transfer you an additional $0.50.

○ I believe people in the study were MORE willing to get vaccinated after seeing both pieces of information (compared to when they saw only the first piece of information)

○ I believe people in the study were LESS willing to get vaccinated after seeing both pieces of information (compared to when they saw only the first piece of information)

○ I believe that seeing both pieces of information DID NOT CHANGE the willingness to get vaccinated of people in the study (compared to when they say only the first piece of information)

Page Break

The actual result in the study was that people who also read the second piece of information (about vaccine hunters) were LESS willing to get vaccinated (and less willing to recommend the vaccine to others), compared to those who did not get this piece of information. (Please do not

share this result with anyone who has a prolific-account, in order to preserve the integrity of this study.)

Why do you think people became LESS willing to vaccinate when they learned about vaccine hunters? Please answer with at least 1 and at most 3 sentences below.

____

____

____

____

____

Page Break

How **likely do you think it is that any of the below reasons explain why** people were less willing to get vaccinated against COVID-19 when they learned about vaccine hunters?

Learning about vaccine hunters makes people...

	Very unlikely	Unlikely	Neither unlikely/likely	Likely	Very likely
...think there is no use in trying to get a vaccine	○	○	○	○	○
...care less about getting vaccinated in order to protect their own health	○	○	○	○	○
...care less about getting vaccinated in order to protect other people's health	○	○	○	○	○
...care less about doing "the right thing" (in this case getting vaccinated)	○	○	○	○	○
...think vaccines are more unsafe	○	○	○	○	○
...think it is less risky to get sick from COVID-19	○	○	○	○	○

End of Block: T2: Vaccine hunters

Start of Block: Covid and vaccine status

Have you tested positive for COVID-19 in the past?

- Yes, I have
- No, I have not

Has anyone you know tested positive for COVID-19 in the past?

- Yes, a family member has tested positive.
- Yes, someone in my social network has tested positive.
- No, I do not know anyone who has tested positive.

Page Break

Do you know someone who was very sick, or died, from COVID-19?

	Yes	No
I know someone who died from COVID-19.	○	○
I know someone who was very sick from COVID-19.	○	○

Page Break

Have you gotten at least the first dose of a COVID-19 vaccine?

- Yes
- No, but I have a plan/appointment to get a vaccine on a specific day
- No

When did you get your first dose of a COVID-19 vaccine? State mm/dd/yyyy below (if you do not remember exactly, please provide your best guess).

____

End of Block: Covid and vaccine status

Start of Block: Demographics basic

Do you generally think of yourself as a Democrat, Republican, Independent, or something else?

- ○ Democrat
- ○ Republican
- ○ Independent
- ○ Something else

What is your gender?

- ○ Female
- ○ Male
- ○ Something else

---

*

What is your age in years?

____

What is the highest level of education you have completed?

- ◯ Less than High School
- ◯ High School / GED
- ◯ Some college
- ◯ 2 year college degree
- ◯ 4 year college degree
- ◯ Professional degree
- ◯ Masters or Doctorate degree

Which of these best describes your race/ethnicity?

- ◯ White/Caucasian
- ◯ Black/ African American
- ◯ Hispanic/ Latino
- ◯ Asian
- ◯ Native American
- ◯ Pacific Islander
- ◯ Other

What was your total income last year? Take into account all your sources of income, including scholarships, health benefits, fringe benefits, and others. Please note that this is your personal income, not the income of your household.

- Less than $10,000
- $10,000 to $20,000
- $20,001 to $30,000
- $30,001 to $40,000
- $40,001 to $50,000
- $50,001 to $60,000
- $60,001 to $70,000
- $70,001 to $80,000
- $80,001 to $90,000
- $90,001 to $100,000
- Over $100,000

In which state do you currently reside?

▼ Alabama ... I do not reside in the United States

End of Block: Demographics basic
